# Oil-Water Separation of Electrospun Cellulose Triacetate Nanofiber Membranes Modified by Electrophoretically Deposited TiO_2_/Graphene Oxide

**DOI:** 10.3390/polym10070746

**Published:** 2018-07-05

**Authors:** Saba Naseem, Chang-Mou Wu, Ting-Zhen Xu, Chiu-Chun Lai, Syang-Peng Rwei

**Affiliations:** 1 Department of Materials Science and Engineering, National Taiwan University of Science and Technology, Taipei 10607, Taiwan; sabanaseem90@gmail.com (S.N.); peggy34389@gmail.com (T.-Z.X.); 2 Department of Textile Engineering, Chinese Culture University, Taipei 11114, Taiwan; lqj2@ulive.pccu.edu.tw; 3 Research and Development Center for Smart Textile Technology, National Taipei University of Technology, Taipei 10608, Taiwan; f10714@ntut.edu.tw

**Keywords:** cellulose triacetate, superhydrophilic, titanium dioxide, graphene oxide, oil-water separation

## Abstract

Recycled waste industrial cellulose triacetate (TAC) film, which is one of the key materials in polarizers, was used to produce nanofiber membranes by electrospinning and synergistic assembly with graphene oxide (GO) and titanium dioxide (TiO_2_) for oil-water separation. In this study, GO and TiO_2_ coated by an electrophoretic deposition method introduced super hydrophilicity onto the recycled TAC (rTAC) membrane, with enhanced water permeability. The results indicate that when the outermost TiO_2_ layer of an asymmetric composite fiber membrane is exposed to ultraviolet irradiation; the hydrophilicity of the hydrophilic layer is more effectively promoted. Moreover, this coating could efficiently repel oil, and demonstrated robust self-cleaning performance during the cycle test, with the aid of the photocatalytic properties of TiO_2_. The rTAC membrane of networked hydrophobic fibers could also increase the speed of the filtrate flow and the water flux of the oil-water emulsion. The permeate carbon concentration in the water was analyzed using a total organic carbon analyzer. Incorporation of TiO_2_/GO onto the rTAC membrane contributed greatly towards enhanced membrane hydrophilicity and antifouling performance. Therefore, the novel TiO_2_/GO/rTAC asymmetric composite fiber has promise for applications in oil-water separation.

## 1. Introduction

With the growth of the world’s population and the advancement of science and technology, the total amount of water used worldwide has increased yearly. There is an increasing percentage of oil wastewater, and industrial wastewater is one of the major sources of water pollution, with varying degrees of color and odor, high biological oxygen demand, and high chemical oxygen demand. The other sources are the metal, food, and textile industries; domestic and agricultural wastewater; and frequent oil spill accidents, which cause severe harm to environmental and human health [1]. In addition to oil pollution, which is more widely known, water may be polluted by the mixture of oil and water produced by crude oil extraction [2]. According to Douglas-Westwood, about 240 million barrels of oil-water mixtures are produced every day. By the year 2020, this is likely to reach 290 million barrels per day [3]. Owing to the large amounts of wastewater produced by industrial and daily household use, oil-water separation has become a global challenge, especially with respect to emulsified oil and water mixtures [4]. In comparison with other technologies such as centrifugation, air flotation, magnetic separation, adhesion, gravity separation, chemical and biological treatment [5,6,7], membrane separation is the most efficient approach for the treatment of oily wastewater, owing to its high separation efficiency, environmental friendliness, and dependability on simple operations [8,9,10]. Special wettability surfaces with porous materials have been designed for effective oil-water separation with antifouling, ultraviolet (UV)-blocking, translucent, self-healing, photocatalytic and anti-bacterial properties [11,12,13,14,15]. Both superhydrophobic–underwater superoleophilic (“oil-removing” type) and superhydrophilic–underwater superoleophobic (“water-removing” type) surfaces have generated interest. Oil-removing-type membranes filter oil droplets through the membrane such that oil adheres to or is adsorbed on it, which decreases efficiency and membrane life [16]. By contrast, the water-removing-type surfaces have several advantages that make them an attractive approach to achieve high-separation efficiency, reduce oil fouling, and easy recycling. However, the performance of the membrane is limited owing to the instability of the materials, because of surfactant adsorption and pore clogging, which causes a decline in efficiency. This remains the limiting factor that restricts the applications of membrane technology. Therefore, there is an urgent need to develop novel membranes to meet the practical requirements.

Cellulose triacetate (CTA) is a polymer produced by the reaction of natural cellulose with acetic anhydride. The degree of substitution of hydroxyl (-OH) on cellulose with carboxyl (-COOH) is 2.7–3.0. Owing to its high transparency, good solvent resistance, and heat resistance, TAC is widely used—in a ratio of 85% CTA to 15%—to make TAC film, which is the main component of polarizers. In general, liquid crystal display monitors use two polarizers, each of which requires two TAC films for protection [17,18,19]. Thus, a large number of polarizers and TAC films are used and become waste. Methods to recycle and repurpose the TAC waste have been discussed in recent years [20]. Here, we recycled waste TAC film to prepare different membranes. The recycling and reusing of TAC film (referred to as rTAC) is an urgent issue. Electrospinning technology can re-dissolve these films to produce nanometer-scale fiber membranes in a simple and straightforward manner. In recent years, electrospinning technology has been studied widely [21,22], and research has been conducted to change the structure of the fiber to make porous and nonporous fibers. This is an initial step towards developing rTAC membranes from industrial-grade TAC film waste, using electrospinning technology combined with the concepts of mixed solvents and steam-induced phase separation to prepare electrospun fibers with porous and non-porous structures [23,24]. The key factor for the formation of membranes and pores is the volatilization rate. Furthermore, different electrospun fiber membrane structures can be prepared [25,26].

Antifouling is closely related to photocatalytic activity and helps to prevent membrane fouling [27]. Nevstrueva et al. [28] reported a TiO_2_/cellulose membrane for an ultra-filtration process and showed that 10 nm TiO_2_ nanoparticles provided good antifouling properties compared with those of larger-sized particles. Chao et al. [29] assembled a graphene oxide (GO)–TiO_2_ film by vacuum filtration and demonstrated its response to additional amounts of dye molecules, indicating that it had potential applications in water purification. Based on the literature, it appears that the membrane surface needs to be modified by incorporation of TiO_2_ to achieve excellent water affinity, by imparting hydrophilicity and helping to wash away the oily parts of oil-water emulsions [30,31]. TiO_2_ is also a material with photocatalytic activity, which can be utilized for the degradation of organic pollutants [32,33]. The potential of TiO_2_ nanomaterials to be reinforced with super hydrophilicity, their self-cleaning surfaces under UV irradiation, high reactivity, mechanical flexibility, non-toxic nature, and low cost, have led to them being regarded as promising candidates for oil-water separation [34,35]. As an innovation, GO has attracted much attention in academic research owing to favorable properties such as its large surface area, and the fact that it is a non-conductive, highly hydrophilic carbon material [36,37]. GO emerged as a good support to achieve uniform distribution without aggregation, and is a promising candidate for oil-water separation [38,39]. GO with a two-dimensional structure has the ability to intercalate into a rTAC fiber membrane and flatten the surface to avoid the penetration of TiO_2_ nanoparticles and prevent blockage of the fiber membrane. After oxidation treatment, GO retains the layered structure of graphite, and many hydrophilic functional groups are introduced onto each monolayer of graphene. Owing to the high hydrophilicity of GO [40], it is highly desirable to fabricate a unique composite layer membrane that can tackle the aggregation of nanoparticles; the synergistic effects of TiO_2_/GO result in higher flux for oil-water emulsions compared with that of a TiO_2_/rTAC membrane without the GO coating. GO also favors photocatalytic activity of TiO_2_ in slowing down e−/h+ recombination and increasing the charge transfer rate [41]. In this research, TiO_2_ and GO were utilized as novel coating materials in electrophoretic deposition (EPD) to modify rTAC membranes, resulting in improved hydrophilicity and minimization of oil adhesion onto the membrane surface. To the best of our knowledge, this is the first time an asymmetric composite TiO_2_/GO/rTAC fiber has been reported and its potential in oil-water separation explored.

## 2. Materials and Methods

### 2.1. Materials

The materials used include titanium (IV) *n*-butoxide (TBOT; 99%, ACROS, Waltham, MA, USA), GO (ACROS), ethanol (EtOH; 99.5%, Jingming Chemical, Miaoli, Taiwan), hydrochloric acid (HCl; 100%, AENCORE, Surrey Hills, Australia), *N*,*N*-dimethylformamide (DMF; 99.8%, ACROS), hexadecane (Sigma-Aldrich, Saint Louis, MO, USA), methylene chloride (MC; Jingming Chemicals), TAC film (FNY, Fuji TD, Tokyo, Japan, thickness 60 µm), and a platinum electrode piece (3 × 2 cm^2^). All reagents were reagent grade and used as purchased.

### 2.2. Synthesis of TiO_2_

TiO_2_ nanoparticles were synthesized via a hydrothermal method similar to that described by Yang et al. [42]. TBOT (15 mL) was mixed with 45 mL of EtOH. After stirring for 30 min, 1 mL of concentrated HCl was added dropwise to the above mixture. The clear solution was transferred to a Teflon-lined stainless steel autoclave and heated at 180 °C for 36 h, followed by cooling to room temperature. The resultant product was collected and centrifuged several times in alcohol and distilled water. The final product was dried at 60 °C.

### 2.3. Electrospinning of rTAC Solution

For porous nanofibers, 5 wt % rTAC was dissolved in a solvent mixture of MC and DMF at a ratio of 9:1 (*v*/*v*) [43]. Non-porous nanofibers were produced with a solvent ratio of MC and EtOH of 9:1 (*v*/*v*) [24]. TAC film (0.5 g) was dissolved in 8.55 g of MC solvent, and 0.95 g of the secondary solvent (DMF/EtOH) was added according to the mixed solvent system used, followed by stirring at 60 rotations per minute. The precursor was transferred into a plastic syringe with a stainless needle (0.31 mm inner diameter) connected to a power supply (KDS 100 Series, KD Scientific, Holliston, MA, USA). The distance between the needle tip and collector was 15 cm, and a voltage of 15 kV was applied. The flow rate of the solution was controlled by a syringe pump and maintained at 0.4 mL/h. All electrospinning solutions were kept at room temperature. Porous and non-porous nanofibers were prepared.

### 2.4. Electrophoresis of GO-rTAC

EPD of GO was carried out directly in the aqueous suspension at room temperature. A 1 wt % aqueous solution containing 0.8 g GO was ultrasonicated for 15 min and further sonicated in an ultrasonic cell disruptor for 1 min. GO-rTAC was obtained by an EPD process using GO aqueous solution as an electrolyte. A pre-cleaned conductive platinum electrode was sandwiched with 18.3 g/m^2^ rTAC electrospun fibrous membrane and clamped in an alligator clip as an anodic electrode. These two platinum electrodes were then slowly immersed 0.5 cm apart in the electrolyte with an applied voltage of 30 V for 15 min. The coated sample was taken out carefully from the EPD and dried horizontally at room temperature [44].

### 2.5. Electrophoresis of TiO_2_ on GO/rTAC

A 0.5% aqueous solution was formed with 0.4 g TiO_2_, and the mixture was ultrasonicated for 15 min before being further sonicated in an ultrasonic cell disruptor for 1 min. TiO_2_/GO/rTAC was obtained by an EPD process using a platinum electrode sandwiched with GO/rTAC, fixed as a cathodic working electrode [45,46]. Thereafter, both the cathode and anode electrode were immersed 0.5 cm apart in the TiO_2_ electrophoresis solution with an applied voltage of 30 V for 15 min. The coated sample was taken out carefully from the EPD and dried horizontally at room temperature. Similarly, EPD was carried out at an applied voltage of 50 V for comparison.

### 2.6. Preparation and Separation of the Oil-in-Water Emulsions

Different stabilized oil-in-water emulsions were prepared by mixing 1.5 g of *n*-hexadecane in 1 L of distilled water and ultrasonically shaking for 30 min. Other oil-in-water emulsions were mixed with 200 mg sodium dodecyl sulfate (SDS) as a surfactant, 1.5 g *n*-hexadecane, and 1 L of distilled water, followed by stirring to produce a milky suspension. Separation was performed using a vacuum filter apparatus fitted with the membrane in a 25 mm glass filter; 20 mL of each oil-in-water emulsion was poured into the glass filter under a suction motor. The filtrate was obtained in the container after the separation process, and its total organic carbon (TOC) rate was determined. This test was conducted for three of the samples by changing the rTAC membrane for porous (rTAC-p), non-porous (rTAC-n), and both non-porous/porous (rTAC-np) nanofiber membranes.

### 2.7. Characterizations

Crystallographic information was obtained by X-ray diffraction (XRD; D2 Phaser X-ray Diffractometer, Bruker, Karlsruhe, Germany). The morphology of rTAC was characterized using high-resolution field emission scanning electron microscopy (FESEM; JEOL JSM-6500F, Tokyo, Japan), which was also employed for characterizing GO/rTAC and TiO_2_/GO/rTAC produced via EPD. Raman spectroscopy was performed to identify GO and GO/rTAC after electrophoresis. The particle size distributions of oil-in-water emulsions and zeta potentials of GO and TiO_2_ suspensions were analyzed by Zetasizer (Malvern Nano-ZS, Worcestershire, UK). H_2_O droplets directly poured onto the sandwiched membrane surface were tested using a contact angle analyzer (Model 100SL, Sindatek, New Taipei, Taiwan). The oil concentrations in the filtrate were ascertained with a total carbon analyzer (Shimadzu C-VCPH, Kyoto, Japan).

## 3. Results and Discussion

### 3.1. XRD

**Figure 1:**
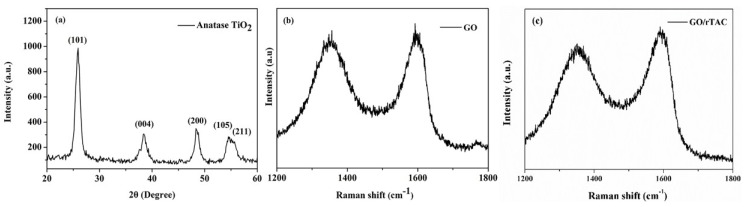
(**a**) X-ray diffraction pattern of TiO_2_ nanoparticles; Raman spectra of (**b**) pure graphene oxide (GO) and (**c**) GO/rTAC after electrophoretic deposition, shown for comparison.

In this work, we chose TiO_2_ nanoparticles synthesized hydrothermally in their anatase phase for the fabrication of the hydrophilic surface. This is because TiO_2_ nanoparticles tend to maintain excellent oil-water separation ability, photocatalytic properties, and mechanical flexibility. It is understood that anatase has higher photocatalytic activity. Figure 1a shows the diffraction peaks that can be attributed to the anatase structure of TiO_2_. All diffraction peaks are well defined and are identified to the corresponding (101), (004), (200), (105) and (211) crystal planes according to the JCPDS 78-2486.

### 3.2. Raman Analysis

Raman spectroscopy was carried out for pure GO and GO after electrophoresis and used to compare GO/rTAC with GO [47]. Figure 1b,c displays the Raman spectroscopy results for pure GO and GO/rTAC in the range of 1200–1800 cm^−1^. Raman shifts for GO and GO/rTAC after electrophoresis were found as 1353 cm^−1^ and 1590 cm^−1^, forming two distinct peaks corresponding to the D band and G band of graphene, respectively. The D band mainly represents the carbon sp^3^ mixture, which results from a defect in the carbon chain, and an oxygen-containing functional group. The G band represents the sp^2^ mixture of carbon. In general, the I_D_/I_G_ ratio represents the degree of internal defects. The original GO I_D_/I_G_ value was 0.99, compared with 0.88 for GO/rTAC after EPD, indicating that the electrophoresis process might have reduced a small fraction of GO to reduced GO, but still exhibited hydrophilicity which was proved by water contact angle result (discussed in Section 3.4. Contact Angle Analysis).

### 3.3. Surface Morphology

The difference in fiber morphology of electrospun rTAC fiber membranes was induced by different types of solvents [48]. Phase separation and volatility of the solvent determines the surface morphology of electrospun nanofibers. To examine the effect of different solvents on surface morphology, a precursor solution was prepared with a solvent ratio of 9:1 (*v*/*v*). The first layer of each oil-water separation film was an electrospun rTAC fiber membrane. In the electrospinning process, the surface area of the liquid column rapidly increases because of the stretching effect of the electric field, which accelerates the solvent evaporation to cause phase separation. Phase separation yields a polymer-rich phase and a solvent-rich phase. When highly volatile solvents are used, fast volatilization occurs. This rapid volatilization causes the concentration of the spinning solution to rise, causing the glass transition to rise and the fiber to rapidly solidify, leaving the solvent-rich phase transformed into pores [49].

**Figure 2:**
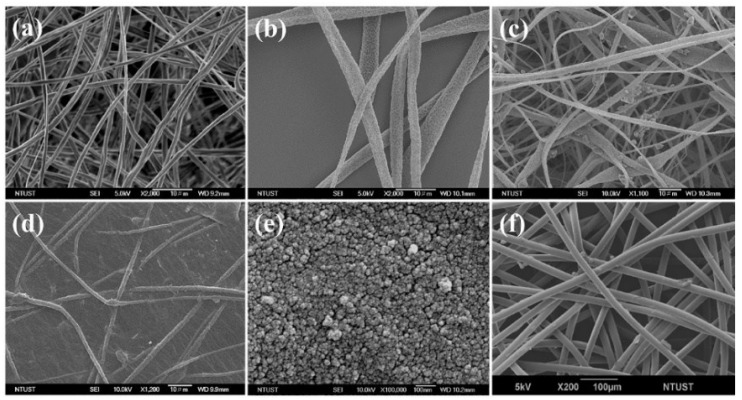
Field emission scanning electron microscopy images showing the morphology of (**a**) rTAC-nonporous fiber; (**b**) rTAC-porous fiber; (**c**) TiO_2_/ rTAC-porous; (**d**) GO/rTAC-porous; (**e**) TiO_2_/GO/rTAC-porous; and (**f**) PP nonwoven fiber.

Figure 2a shows an image of the rTAC-n sample, for which the solution was configured as MC: EtOH 9:1 (*v*/*v*). It can be seen that there were no holes in the fibers, and strips can be observed. The fiber had a rough surface texture, presumably because of the phase separation effect in the electrospinning environment at room temperature. As expected, there was a difference in the volatilization rate between the primary and secondary solvents, the gap was obvious enough to produce only rough fibers. Owing to the addition of EtOH, solidification became dominant, resulting in a non-porous fiber.

For the rTAC-porous sample, the solvents were MC and DMF in a 9:1 (*v*/*v*) ratio. The boiling point of MC is 39.6 °C, that of DMF is 153 °C, and that of EtOH is 76 °C. Thus, compared with that of the MC-EtOH solvent systems, there was a large boiling point difference between the primary and secondary solvents in the MC-DMF solvent system, and the secondary solvent remained in the fiber. The reasons for the porous structure within the fibers were that the secondary solvent remained longer in the solvent-rich zone, and the phase separation effect was more obvious. In addition, DMF itself is highly hygroscopic; when the main solvent evaporated, this might have caused the surface temperature of the fiber to drop, leading to condensation of moisture into water droplets and miscibility of water droplets with DMF. Owing to the high water content, water vapor could act as a non-solvent molecule, further hindering the process of dissolution and precipitation between a smooth fiber-type solvent and water molecules so that the pore structure became complete with a certain depth. The FESEM image of rTAC-porous in Figure 2b shows the fiber type of the mixed solvent system of MC and DMF. It can clearly be observed that the fiber had uniform pores with a good morphology.

Figure 2c presents a FESEM image of the TiO_2_/rTAC-porous sample; the third layer of the oil-water separation membrane is an electrophoretic layer of GO/TiO_2_ on rTAC-p. Hydrothermal synthesis resulted in TiO_2_ with a particle size of about 10 nm; however, the rTAC electrospinning pores were larger than 10 nm, allowing TiO_2_ powder to enter the pores of the fibers. As a result, it failed to drape over rTAC and made the surface more uneven, further reducing the hydrophilicity. To solve this problem, GO was coated on the rTAC-p fiber by electrophoresis first, with an applied voltage of 30 V, to smooth and flatten the rTAC fibers with a stabilized suspension (Figure 2d). This was verified by zeta potential analysis of 1 wt % GO aqueous suspension, which gave a value of −43.6 mV. GO deposited on the rTAC membrane, which is favorable for hydrophilicity [50,51] since the functional groups of GO are mostly carboxyl. Carboxylic acids are expected, based on their structure, to be polar, and can be generated with the same or other molecules. Hydrogen bonds and polar molecules can be miscible with water of the same polarity when the surface is flat.

**Figure 3:**
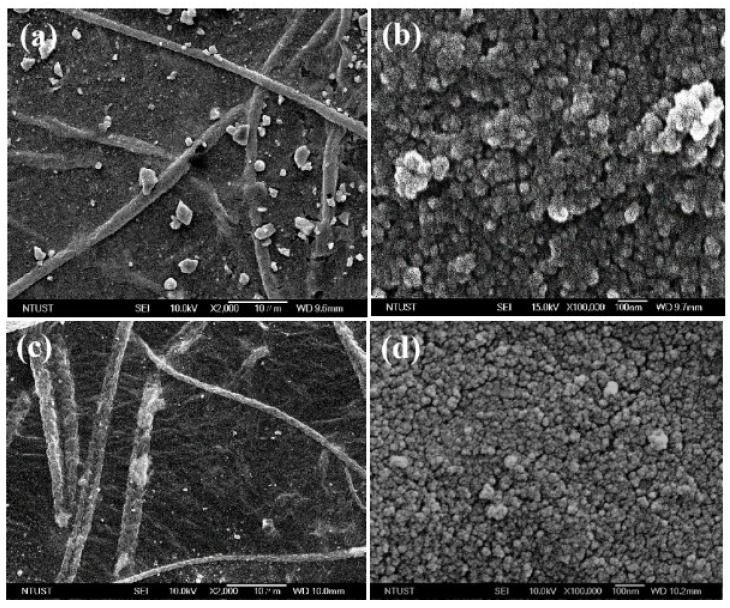
FESEM micrographs of the TiO_2_ nanofilm deposited by electrophoresis on GO/rTAC film: (**a**,**b**) 30 V TiO_2_/30 V GO/rTAC at different magnifications and (**c**,**d**) 50 V TiO_2_/30 V GO/rTAC at different magnifications.

It was easier to obtain a uniform and densely distributed TiO_2_ by electrophoresis on GO/rTAC film, as can be seen in the FESEM image of the TiO_2_/GO/rTAC-p sample in Figure 2e. The zeta potential of the 0.5 wt % TiO_2_ aqueous suspension was 31.7 mV. Figure 3 shows the morphologies of the TiO_2_/GO/rTAC film, optimized by adjusting an applied voltage. Images of the electrophoresis of TiO_2_ on GO/rTAC-p and GO/rTAC-n with applied voltages of 30 V and 50 V are shown in Figure 3a–d. The nanofilm TiO_2_ particles densely covered the GO/rTAC film at 50 V, presumably because of the high voltage; increased migration rates of TiO_2_ particles were revealed in the FESEM images compared with those at 30 V. The FESEM images of TiO_2_/30V GO/rTAC (Figure 3a,b) at 30 V show that the film was not well coated with TiO_2_ particles, which attached to the GO surface only. As the applied voltage increased to 50 V, the TiO_2_ particles deeply and evenly coated the surface of the GO/rTAC film, as can be seen in the FESEM image in Figure 3c. Overall, the rate of formation of the TiO_2_ layer increased with the applied voltage, and consequently, the thickness of the TiO_2_ layer increased, as is evident in the higher-magnification FESEM image shown in Figure 3d.

The supporting layer for the oil-water separation was a commercially available spun-bonded non-woven fabric. The main advantages of non-woven fabrics are that they are breathable, soft, easy to sew, fold-resistant, insulated, rainproof, leak-proof, tough, strongly absorbent, and low cost. Figure 2f shows a polypropylene nonwoven fiber as a standing layer of oil-water separation film.

### 3.4. Contact Angle Analysis

**Figure 4:**
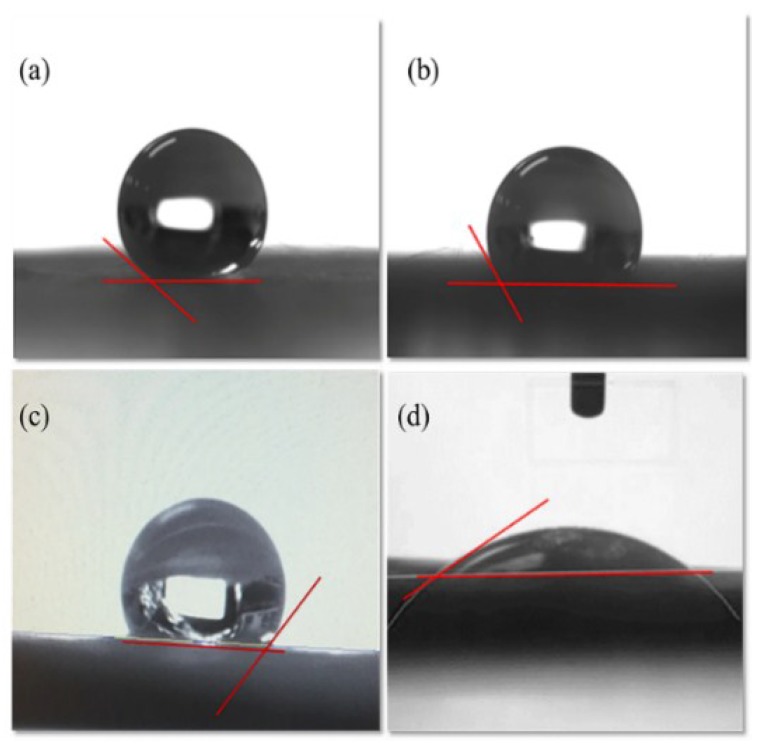
Contact angles of water droplets in air on different samples: (**a**) rTAC-p; (**b**) rTAC-n; (**c**) TiO_2_/rTAC-p; and (**d**) GO/rTAC-p.

**Figure 5:**
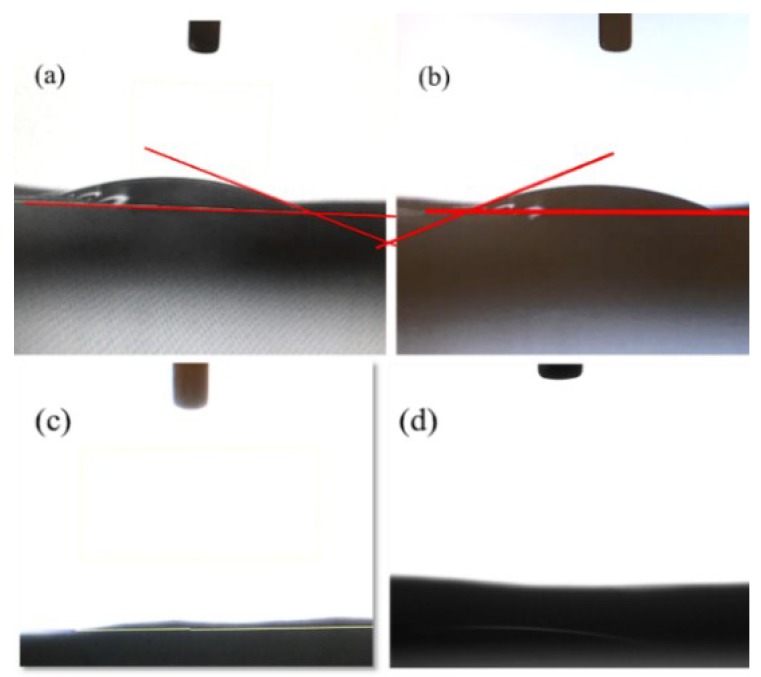
Water contact angle in air on different samples: (**a**) TiO_2_/GO/rTAC-p; and (**b**) TiO_2_/GO/rTAC-n; WCA in air after UV irradiation for 1 h: (**c**) TiO_2_/GO/rTAC-p; and (**d**) TiO_2_/GO/rTAC-n.

The contact angle depends on interactions with three different interfaces. For a hydrophobic surface, the contact angle should be greater than 90°. For superhydrophobic surfaces, contact angles should be as high as 150° or even approach 180°. For the solid surface to be hydrophilic, contact angles should be less than 90°, and for superhydrophilic surfaces, the angle is defined as less than 10°. The results of water contact angle measurements in air on different samples are illustrated in Figure 4a–d; the contact angle of rTAC-porous was 140°, which could be a “oil-removing” type surface. However, for rTAC-porous, it was just a hydrophobic fiber membrane and showed a relatively weak superhydrophobic property which could not meet the requirement for highly efficient oil-water separation. Natural cellulose has many hydrophilic groups, but cellulose triacetate is an ester-substituted polymer. The presence of hydrophilic groups alone is not sufficient to render it hydrophilic, hence its hydrophobic character. The rTAC-non-porous sample can be seen in Figure 4b with a contact angle of 117°; the structure of rTAC-n was the same as that of rTAC-p, but its surface was smoother and its fiber diameter was less. In general, both rTAC-p and rTAC-n are hydrophobic fibers, but rTAC-p has larger antennae than rTAC-n. The hydrothermal synthesis of TiO_2_ resulted in a particle size of about 10 nm; however, rTAC-p had pore sizes larger than 10 nm, allowing the TiO_2_ powder to enter the fibrous pores. This meant that the rTAC-p surface could not be even, and the hydrophilicity was reduced. The contact angle of TiO_2_/rTAC-p did not reach the threshold required for hydrophilicity, i.e., it was found to be 124°, as shown in Figure 4c. However, after electrophoretic deposition of GO on rTAC-p, the contact angle of GO/rTAC was decreased owing to the dense deposition and reliance on GO itself. The COOH group caused the contact angle to drop to 31°, as shown in Figure 4d. Clearly, the electrophoretic TiO_2_ layer was very dense. As illustrated in Figure 5a–d, the water contact angle in air after UV irradiation was rapidly boosted. Figure 5a,b show that the contact angle of a water droplet on TiO_2_/GO/ rTAC-p (Figure 5a) and TiO_2_/GO/rTAC-n (Figure 5b) varied from approximately 17° to 22°. The measurements varied slightly for different areas. As shown in Figure 5c,d, after exposure to UV illumination, the water contact angles on both TiO_2_/GO/rTAC-p and TiO_2_/GO/rTAC-n were <5°, indicating superhydrophilic character [52,53]. This is because irradiation with UV light allows the surface of TiO_2_ to produce oxygen vacancies at bridging sites and, simultaneously, the electrons to reduce Ti^4+^ to Ti^3+^ with the trapping of holes close to the surface of the semiconductor, thus weakening the bonds between titanium and oxygen, and creating vacancies, which increases the hydrophilicity [54]. CTA polymer might degrade under UV radiation and prolonged heating [55]. However, in this study, UV irradiation was only for 1 h and also without heating. Besides, the radiations might be absorbed by TiO_2_ and GO layers. Therefore, the CTA degradation problem on the studied membranes could be avoided and, therefore, not affect its service life.

**Figure 6:**
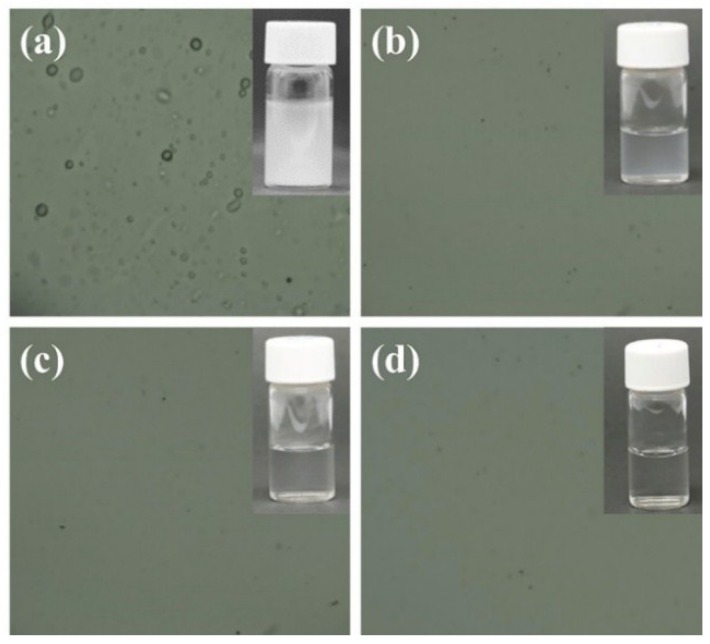
Demonstration of permeate: (**a**) oil-water; (**b**) TiO_2_/GO/rTAC-p; (**c**) TiO_2_/GO/rTAC-n and (**d**) TiO_2_/GO/rTAC-np.

**Figure 7:**
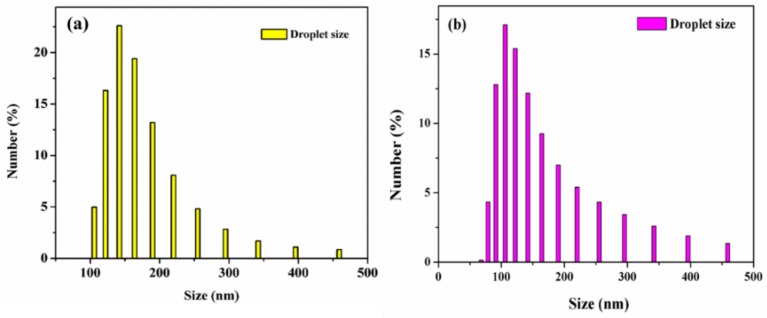
(**a**) Particle size distribution of hexadecane oil in water emulsion in the feed and (**b**) particle size of the SDS-stabilized hexadecane oil in water emulsion in the feed.

The oil was classified as n-hexadecane at a concentration of 1500 ppm. Figure 6 shows a schematic of the filtration of the hexadecane in the water emulsion separation process. As the oil-in-water emulsion was poured onto the samples, hexadecane was repelled by the samples and water quickly permeated through the samples. No oil particles were seen in the permeate solution, owing to superhydrophilicity. Table 1 summarizes the residual TOC rate of hexadecane oil; the water permeating through the TiO_2_/GO/rTAC-p sample had a higher concentration of 29 ppm, while that of the TiO_2_/GO/rTAC-n sample was 22.65 ppm. This was because the fiber diameter of rTAC-n was finer and the pores were also smaller; hence, the filtrate could be obtained at lower concentrations. TiO_2_/GO/rTAC-np had the lowest concentration of 16.92 ppm. This might have been because the two fiber sizes could filter more. As shown in Figure 6, the original oil-in-water emulsion had a milky-white color before treatment, while after treatment the permeate solution was transparent. The optical microscopy images revealed that there was an oil droplet in the feed of the oil-in-water emulsion, but no oil droplets could be seen in the permeate. The particle size distribution of the oil-in-water emulsion in the feed had a mean size of 100–200 nm (Figure 7a). TiO_2_/GO/rTAC-np performed better than TiO_2_/GO/rTAC-p or TiO_2_/GO/rTAC-n. TiO_2_/GO/rTAC-np was subjected to a cycle experiment. Two hours after irradiation of the contaminated sample with UV light, the organic material was photodegraded and the sample recovered was found to be superhydrophilic. After filtration, the TiO_2_/GO/rTAC-np obtained with the residual oil was present at a concentration of 26.48 ppm. Although the concentration slightly increased, this might have been because some oil particles were trapped in and filled the voids, but still maintained the ability to filter grease. The photocatalytic degradation of organic material endows TiO_2_/GO/rTAC-np with an antifouling and self-cleaning ability even after being contaminated with oil. The oil rejection coefficient was calculated by Equation (1).

(1) $$R=\left(1-\frac{C_{p}}{C_{o}}\right)\times 100\%$$

where C_o_ and C_p_ are the concentrations of oil in the original oil-in-water emulsions and in the permeate solution, respectively. All samples achieved a high oil rejection coefficient for the original hexadecane oil in water: 98.9% for TiO_2_/GO/rTAC-np, 98.5% for TiO_2_/GO/rTAC-n, and 98.0% for the TiO_2_/GO/rTAC-p. An oil-in-water emulsion stabilized with the surfactant SDS was prepared with the same concentration of 1500 ppm. The particle size of the SDS-stabilized oil-in-water emulsion in the feed was less than 100 nm (Figure 7b). As shown in Table 1, the residual TOC rate of hexadecane oil in the permeate was lower than that in the feed. The concentration of the TiO_2_/GO/rTAC-np sample in the permeate was still 177.2 ppm, but the concentrations of TiO_2_/GO/rTAC-p and TiO_2_/GO/rTAC-n were 235.9 ppm and 235.5 ppm, respectively. This was an expected result, as the oil droplet particle sizes were decreased by the surfactant and thus they could permeate through the membranes. Moreover, the oil rejection coefficients for SDS-stabilized hexadecane oil in water were lower compared with those for the original oil in water for all samples, but still convincing, at 88.2% for TiO_2_/GO/rTAC-np, 84.3% for TiO_2_/GO/rTAC-n, and 84.3% for TiO_2_/GO/rTAC-p. Despite the high TOC value in the permeate, the result remained consistent with that for the surfactant-free oil-in-water emulsion. Among all the samples, both non-porous/porous rTAC nanofiber membranes achieved an excellent oil rejection coefficient for both original hexadecane oils in water and SDS-stabilized hexadecane oil in water, indicating effective oil-water separation performance.

## 4. Conclusions

In this research, we have successfully developed three-layered TiO_2_, GO, and rTAC asymmetric composite fiber membranes for oil-water separation. The first step was to prepare electrospun rTAC fiber membranes to provide a smooth passage for the filtrate. We successfully recycled and reused waste TAC film to meet the demands of development and to ensure environmental friendliness and high-value application. The second step was the electrophoresis of GO on the rTAC fiber membrane, which introduced hydrophilicity and smoothly flattened the surface. This was followed by electrophoretic deposition of TiO_2_ on the GO/rTAC film. With the aid of UV light, sufficient oxygen vacancies were generated to allow the contact angle to drop below 5°, effectively enhancing the hydrophilicity of the hydrophilic layer. The resulting both non-porous/porous rTAC nanofiber membranes exhibited high oil rejection coefficients of 98.9% and 88.2% for the surfactant-free and surfactant-stabilized oil-water emulsions, respectively. The non-porous/porous rTAC nanofiber membranes successfully and effectively achieved the goal of oil-water separation with antifouling and self-cleaning properties, indicating a potential for applications in removing emulsified oil, which would benefit the environment, and human health.

## Figures and Tables

**Table 1:** Residual TOC rates of raw n- hexadecane oil and SDS-stabilized n-hexadecane oil emulsion in permeate for different samples.

Samples	Raw n-Hexadecane Oil (ppm)	SDS Stabilized n-Hexadecane Oil Emulsion (ppm)
Oil in feed	1500	1500
TiO_2_/GO/rTAC-p	29.00	235.9
TiO_2_/GO/rTAC-n	22.65	235.5
TiO_2_/GO/rTAC-np	16.92	177.2
